# Sustainable Water Remediation Through Degradation of Organic Pollutants Using Green‐Synthesized Cerium and Sulfur‐Doped TiO_2_ Nanoparticles

**DOI:** 10.1002/cbdv.71765

**Published:** 2026-10-06

**Authors:** Fahim Ullah, Jafar Saifullah, Sufian Rasheed, Saima Gul, Safina Sharif, Syed Shakil Abbas, M. I. Khan

**Affiliations:** ^1^ Department of Chemistry Kohat University of Science & Technology Kohat Khyber Pakhtunkhwa Pakistan; ^2^ HEJ Research Institute of Chemistry International Center for Chemical and Biological Sciences (ICCBS) University of Karachi Karachi Sindh Pakistan; ^3^ Gomal Centre for Biochemistry & Biotechnology Gomal University Dera Ismail Khan Khyber Pakhtunkhwa Pakistan

**Keywords:** green synthesis, organic pollutant degradation, photocatalysis, reusability, titania nanoparticles

## Abstract

Eco‐friendly photocatalysis offers a sustainable approach for degrading hazardous water pollutants. In this study, sulfur‐doped (S─TiO_2_) and cerium‐doped (Ce─TiO_2_) nanoparticles were synthesized via a green route using *Syzygium cumini* L. leaf extract. Comprehensive characterization via SEM, FTIR, UV–vis diffuse reflectance spectroscopy, photoluminescence (PL), Raman, and XPS confirmed successful dopant incorporation, introduced mid‐gap defect states, narrowed the band gap (2.0–2.5 eV), and promoted efficient charge separation. Photocatalytic performance was optimized across varying parameters including catalyst loading, solution pH, and contact time. Under optimized conditions, S─TiO_2_ nanoparticles achieved degradation efficiencies of 98.5% for Rhodamine B (RhB) within 90 min, 96.0% for omeprazole (OMZ) within 30 min, and 98.1% for chlorpyrifos (PCPS) within 40 min, slightly outperforming Ce─TiO_2_ (97.3%, 95.55%, and 97.3%, respectively). Radical scavenger studies confirmed •OH and •O^2−^ as the primary reactive species driving degradation. Reusability evaluations demonstrated exceptional structural and chemical stability over successive cycles with minimal activity loss. Benchmarked against literature standards, these green‐synthesized nanostructures present robust potential for practical wastewater remediation applications.

## Graphical abstract

Cerium‐ and sulfur‐doped titanium dioxide nanoparticles were synthesized adopting a green route using *Syzygium cumini* L. leaf extract leading to narrowing the band gap (2.0–2.5 eV). Optimization of photocatalysis was achieved by varying parameters including catalyst loading, pH, contact time, reusability study, and chemical stability of target organic pollutants including Rhodamine B, omeprazole, and chlorpyrifos. The reported materials exhibited potential for practical wastewater remediation applications when compared with the literature.

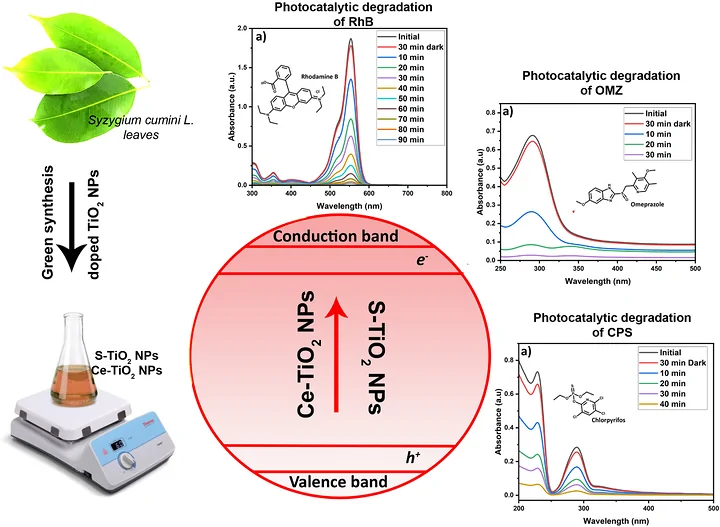

## 1 Introduction

Water pollution is a growing environmental challenge due to its harmful effects on ecosystems and human health. A significant contributor is the discharge of contaminants into water bodies, which threatens water quality and the organisms that depend on it [1, 2]. Dyes, widely used in the textile, paper, and leather industries, are particularly problematic. RhB, a synthetic dye frequently found in wastewater, impairs water quality and risks aquatic life and human health [3, 4, 5]. Its persistence and toxicity make its removal crucial [6]. Pesticides, widely used in agriculture, also contribute to water pollution. CPS, an organophosphate pesticide, protects crops but can run off into water bodies, harming aquatic organisms and ecosystems [7, 8]. Its toxicity and potential for bioaccumulation raise long‐term environmental concerns [9]. Pharmaceuticals are another class of contaminants, with OMZ, a proton pump inhibitor, being; OMZ, a proton pump inhibitor, has been detected in water sources [10]. Pharmaceuticals in water pose ecological risks and may contribute to antibiotic resistance [11]. The toxic effects of these pollutants are well‐documented [12, 13]. For example, RhB affects aquatic organisms like fish and algae, while CPS poses risks to non‐target species such as birds and aquatic invertebrates [14]. Pharmaceuticals can also disrupt endocrine systems in aquatic life and pose risks to human health [15] (Figure 1).

**FIGURE 1:**
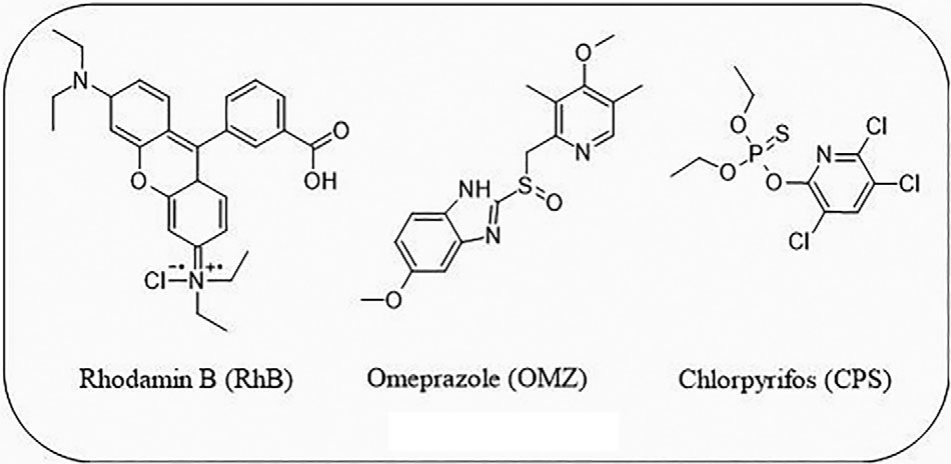
Chemical structures of RhB, OMZ, and CPS.

Various methods have been explored to address water pollution, including physical, chemical, and biological processes, each with advantages and limitations. Advanced oxidation processes (AOPs) have gained attention for effectively degrading organic pollutants [16]. Photocatalysis, a subset of AOPs, uses semiconductor materials to generate reactive oxygen species (ROS) under light, breaking pollutants into harmless by‐products like carbon dioxide and water [17, 18]. This method offers the advantage of using clean, renewable energy sources such as solar or artificial light, making it an attractive solution for treating water contaminated with dyes, pesticides, and pharmaceuticals [19, 20]. The choice of photocatalyst is crucial for process efficiency [21, 22, 23]. TiO_2_, ZnO, and other metal oxides have been widely studied, with TiO_2_ gaining significant attention due to its high reactivity, chemical stability, and low cost [24]. However, the wide bandgap of pristine TiO_2_ limits its use of solar light, prompting modifications to enhance visible light activity [25].

Nanoscale TiO_2_, in particular, has shown promise due to its high surface area and unique optical properties, which improve photocatalytic efficiency by providing more active sites and enhancing light absorption [26]. Consequently, nanoscale TiO_2_‐based photocatalysts are extensively researched for treatment applications [27]. However, conventional nanomaterial synthesis often involves hazardous solvents and energy‐intensive processes, raising environmental and sustainability concerns, thereby highlighting the need for eco‐friendly and sustainable synthesis methods [28, 29].

Green synthesis, also known as environmentally benign synthesis, offers a promising alternative to traditional chemical methods for producing nanomaterials [30, 31]. This approach uses non‐toxic precursors, environmentally friendly solvents, and energy‐efficient processes to fabricate nanomaterials with controlled size, shape, and composition [32]. Green synthesis aims to minimize the generation of hazardous substances and reduce the environmental impact of chemical processes [33, 34, 35]. The benefits of green synthesis for producing nanomaterials are manifold; by utilizing natural sources such as plant extracts, microorganisms, and biopolymers as reducing and stabilizing agents offer a sustainable and cost‐effective route for the fabrication of nanomaterials [36]. Using renewable resources and avoiding toxic chemicals in the synthesis process help reduce environmental pollution and conserve energy and natural resources. Furthermore, green‐synthesized nanomaterials have been shown to exhibit excellent performance in various applications, including photocatalytic degradation of pollutants, highlighting their potential for sustainable development and represent a significant advancement in environmental nanotechnology [37, 38, 39, 40]. The utilization of green‐synthesized nanomaterials for the degradation of specific contaminants holds great promise for addressing water pollution challenges effectively and in an environmentally benign manner. The photocatalytic degradation of individual organic pollutants using doped TiO_2_ is well‐documented; the challenge remains in designing highly efficient, sustainable catalysts capable of managing mixed‐contaminant streams such as dyes, pesticides, and pharmaceuticals simultaneously. Existing methods often rely on hazardous solvents or complex, energy‐intensive synthesis protocols [41, 42, 43, 44].

This study addresses these limitations by reporting the green synthesis of S‐ and Ce‐doped TiO_2_ nanoparticles using *Syzygium cumini* L. leaf extract. The extract is usually rich in bioactive phytochemicals, specifically polyphenols, flavonoids, and hydroxyl groups [35], acting as natural reducing and stabilizing agents to facilitate the eco‐friendly reduction of titanium precursors into stable nanoparticles. This approach combines a non‐toxic synthesis route with a dual‐doping strategy that significantly improves charge separation and visible‐light responsiveness. By investigating the simultaneous degradation of RhB, OMZ, and CPS, we show that our catalysts provide a more versatile and sustainable solution for complex wastewater remediation than previously reported mono‐pollutant studies.

Our findings demonstrate that green‐synthesized S─TiO_2_ and Ce─TiO_2_ NPs exhibit superior degradation efficiencies, highlighting their potential as sustainable photocatalysts for water remediation applications.

## 2 Experimental Section

### 2.1 Reagents

Titanium tetrachloride, ammonium cerium(IV) nitrate and RhB were procured from Merck Millipore. Thiourea and ammonia solution were sourced from Sigma‐Aldrich. Ethanol, hydrochloric acid (HCl), and sodium hydroxide (NaOH) were acquired from Fisher Scientific U.K. Analytical‐grade OMZ and CPS were obtained from local pharmaceutical and pesticide industries (Bayer Crop Science and Pharmaceuticals Pakistan). Deionized water was used throughout the experimental procedures, including nanoparticle synthesis and degradation experiments.

### 2.2 Green Synthesis of Ce and S‐Doped TiO_2_ Nanoparticles

Fresh leaves of *Syzygium cumini* L., commonly known as Java plum or black plum, were collected from the Company Bagh, Kohat, Khyber Pakhtunkhwa, Pakistan, for the green synthesis of TiO_2_ nanoparticles; the leaves were thoroughly washed and shade‐dried. After drying, the leaves were finely ground using a mortar and pestle to obtain a uniform powder (10 g), immersed in deionized water (100 mL), and stirred for 48 h at room temperature to extract the bioactive components. The resultant extract was filtered through Whatman filter paper No. 1 (11 µm pore size) to obtain a clear solution for nanoparticle synthesis.

The synthesis of S─TiO_2_ and Ce─TiO_2_ nanoparticles followed a procedure adapted from the literature with modifications [45, 46]. For both nanoparticles, 100 mL of *Syzygium cumini* L. leaf extract was used. We added 10 mL of titanium tetrachloride (TiCl_4_) dropwise to the leaf extract under continuous stirring at room temperature. For S─TiO_2_ NPs, thiourea 5 wt. % was added dropwise as the sulfur source. For Ce─TiO_2_ NPs, 5 wt.% ammonium cerium(IV) nitrate was used as the cerium source. The 5 wt.% concentration for S and Ce precursors was selected based on preliminary optimization trials. Concentrations below this threshold yielded insufficient band‐gap narrowing, whereas levels exceeding 5 wt.% introduced surface defect clusters that facilitated charge‐carrier recombination, thereby reducing photocatalytic efficiency. The TiCl_4_/leaf extract ratio was approximately 1:10. The solution pH was adjusted to 9 using a dilute ammonia solution to facilitate nanoparticle formation. The mixture was stirred until a visible color change occurred, then aged at room temperature for 24 h to ensure complete nanoparticle formation.

After alkaline hydrolysis of the leaf extract to form an intermediate titanium hydroxide/hydrous oxide precursor, the recovered precipitates were separated via centrifugation, washed thoroughly with deionized water and ethanol to remove soluble impurities, dried at 60°C overnight, and then thermally calcined at 400°C for 3 h to remove residual organics and obtain a highly crystalline anatase phase.

### 2.3 Materials Characterization

The morphology and surface features of the synthesized nanoparticles were examined using a JEOL JSM‐6500F scanning electron microscope (SEM) operated at an accelerating voltage of 30 kV. Powder x‐ray diffraction (PXRD) patterns were recorded on a Bruker D8 Advanced x‐ray diffractometer equipped with Cu‐Kα radiation (λ = 1.5406 Å) operating at 40 kV and 40 mA. Scans were performed over a 2θ range of 20°–80° with a continuous scan rate, and the resulting patterns were matched with the standard JCPDS card (No. 21‐1272) to confirm the crystalline anatase phase of TiO_2_. Fourier‐transform infrared (FT‐IR) spectra were collected using a Bruker Optics ATR‐FT‐IR Platinum instrument over 400–4000 cm^−^^1^ with a resolution of 4 cm^−^^1^. Optical absorbance properties were measured using a Shimadzu UV‐1800 UV–vis spectrophotometer over 200–800 nm using aqueous nanoparticle dispersions. Photoluminescence (PL) emission spectra were acquired on a Drawell DW‐F97 fluorescence spectrophotometer using an excitation wavelength of 320 nm across a spectral range of 350–650 nm. Structural and vibrational properties were investigated using a Renishaw in Via‐Reflex Raman microscope system equipped with a 532 nm solid‐state laser excitation source at ambient temperature. X‐ray photoelectron spectroscopy (XPS) measurements were performed on a Perkin‐Elmer RBD apparatus coupled with an updated PHI‐5300C ESCA system utilizing monochromatic Mg‐Kα radiation (1253.6 eV) to determine the surface elemental composition and oxidation states.

### 2.4 Photocatalytic Assessment

The photocatalytic efficacy of both S─TiO_2_ and Ce─TiO_2_ NPs was evaluated using a dedicated reactor equipped with a glass chamber for RhB, OMZ, and CPS. A xenon arc lamp, positioned 15 cm above the samples, was employed as the radiation source, providing a power output ranging from 75 to 1000 W and emitting a broad spectrum from 240 to 850 nm, with a luminous flux between 20 000 and 500 000 cd/cm^2^. The radiation intensity over the samples was approximately 350 W/M^2^, measured using a radiometer. In this study, no UV filter was employed to isolate the contribution of UV and visible light. Therefore, the photocatalytic tests were performed under broad‐spectrum light, where both UV and visible light contributed to pollutant degradation. TiO_2_ NPs exhibit strong photocatalytic activity under UV light, which likely played a significant role in the degradation process observed in this experiment. Control experiments were also conducted to assess the effect of photolysis by irradiating the solutions under the same conditions without the nanoparticles. These tests allowed us to determine any degradation due to light alone, ensuring that the observed degradation was primarily due to the photocatalytic action of the NPs

The photoreactor components were enclosed within a sealed box during irradiation to minimize photon loss, with a maximum exposure time of 90 min. Before irradiation, the system was shaken in the dark for 30 min to establish adsorption–desorption equilibrium between the solution and the photocatalysts. RhB, OMZ, and CPS degradation were monitored at 10 min intervals using a UV–vis spectrophotometer, with 2 mL samples taken and centrifuged to remove the photocatalyst particles. Degradation parameters, including catalyst loading, pH, and reaction time, were optimized to achieve maximum degradation efficiency to achieve maximum degradation efficiency. The percentage degradation was calculated using the following equation:

$$\%\;\mathrm{degradation}\;=\left(1-\frac{A}{A_{0}}\right)\times 100$$

## 3 Results and Discussion

### 3.1 Chemical and Structural Characterization of S─TiO_2_ NPs and Ce─TiO_2_ NPs

*Syzygium cumini* L. leaf extract plays a vital role in the green synthesis of TiO_2_ nanoparticles, specifically, the aqueous leaf extract contains a rich profile of bioactive secondary metabolites, predominantly phenolic acids (such as gallic acid, ellagic acid, and chlorogenic acid), flavonoids (including myricetin, quercetin, and kaempferol derivatives), and hydrolyzable tannins [47, 48]. These compounds facilitate the eco‐friendly reduction of titanium precursors (Ti^4+^) into TiO_2_ nanoparticles without requiring toxic chemicals. While TiCl_4_ can be converted through hydrolysis and calcination, the reducing agents in the leaf extract promote electron transfer, driving reactions under milder conditions. Specifically, these biomolecules transfer electrons to titanium hydroxide species, promoting the reduction of Ti^4+^ to Ti^3+^ states [47, 48]. The reduced titanium species act as primary nuclei, fostering the formation of TiO_2_ nanoparticles through redox interactions with ambient oxygen [49]. Additionally, the organic molecules in the extract act as efficient capping agents, stabilizing the nanoparticle surfaces, preventing agglomeration, and ensuring uniform nanoscale dimensions. Beyond structural stabilization, the extract facilitates the uniform incorporation of sulfur or cerium dopants into the TiO_2_ lattice, modifying the band gap and significantly enhancing visible‐light absorption capacity [50].

The PXRD patterns of the synthesized materials displayed prominent characteristic peaks perfectly indexed to the tetragonal anatase phase of TiO_2_ NPs (JCPDS Card No. 21‐1272). A slight redshift of the diffraction peaks was observed in the S‐ and Ce‐doped samples, which is ascribed to unit cell expansion and local lattice strain induced by the successful incorporation of larger dopant ions into the TiO_2_ NPs crystal lattice. The specific peaks correspond to various crystal planes of anatase TiO_2_ nanoparticles, such as (101), (103), (004), (112), (200), (105), (211), (204), and (116) [40]. Notably, no significant diffraction peaks attributable to S or Ce were detected in the S─TiO_2_ NPs or Ce─TiO_2_ samples, indicating their low concentrations in the samples [51]. The analysis confirmed the phase purity and crystallinity of the synthesized materials, supporting their potential applications in photocatalysis. The absence of distinct diffraction peaks associated with S and Ce further corroborates the efficacy of PXRD in characterizing materials at the atomic level, thereby providing details about their composition and structural properties, however, subsequent characterization identified S and Ce.

Fourier transform infrared spectroscopy (FTIR) was employed to characterize the functional groups and chemical structures of the prepared TiO_2_, S─TiO_2_, and Ce─TiO_2_ nanoparticles (Figure 2B). The FTIR spectrum of pure TiO_2_ exhibited a prominent peak at approximately 440 cm^−^^1^, corresponding to the Ti─O stretching vibrations in the TiO_2_ lattice [47, 48, 49]. In both S─TiO_2_ and Ce─TiO_2_ samples, this peak broadened and shifted to around 580 cm^−^^1^. This broadening could be attributed to incorporating dopant ions (S or Ce) into the TiO_2_ lattice, potentially affecting the Ti─O bond environment [47, 48, 52].

**FIGURE 2:**
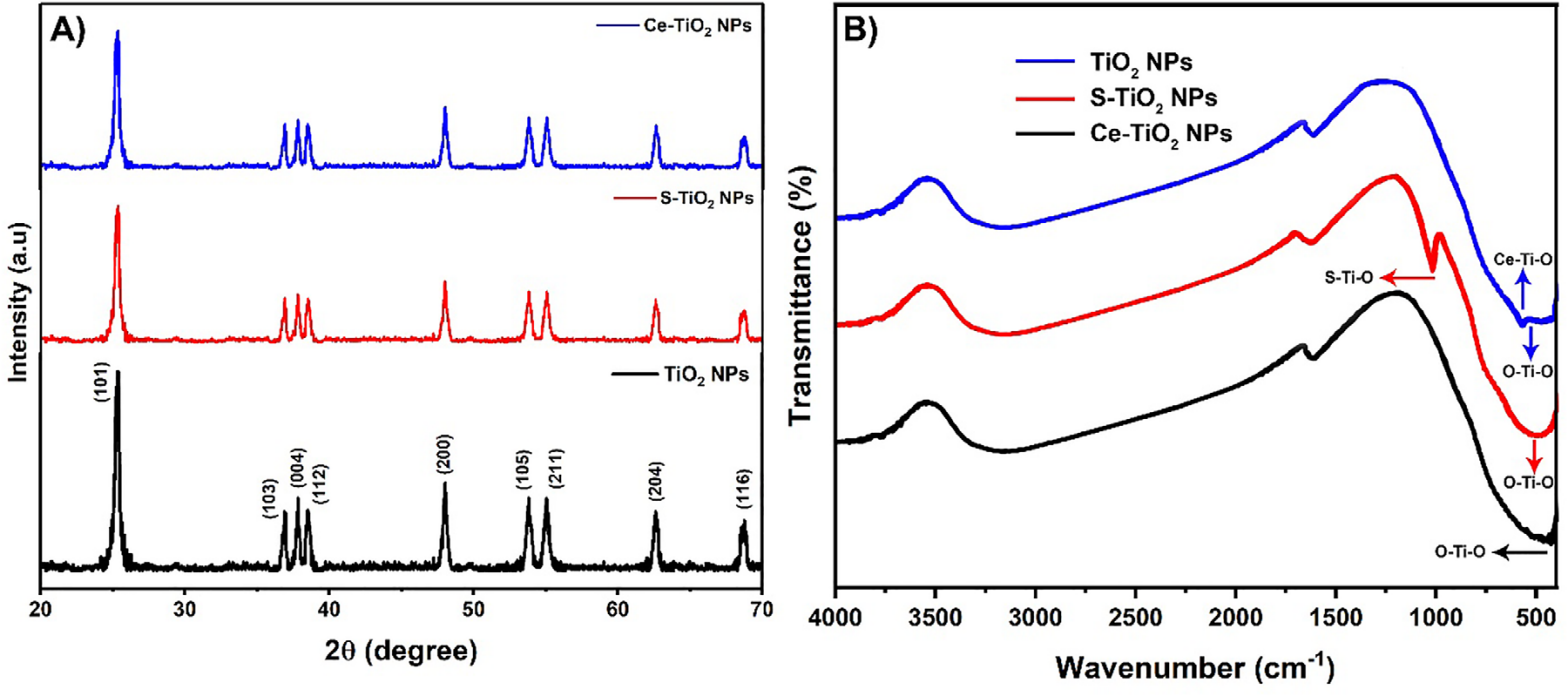
PXRD pattern (A) and FTIR spectra (B) of TiO_2_ NPs, S─TiO_2_ NPs, and Ce─TiO_2_ NPs.

The S─TiO_2_ NPs spectrum displayed an additional peak at 1055 cm^−^^1^. This peak falls within the reported range for Ti─O─S bond vibrations (1000–1100 cm^−^^1^), suggesting the successful introduction of sulfur dopants into the TiO_2_ structure [47, 48, 50]. For the Ce─TiO_2_ NPs, the peak associated with the Ce─Ti─O bond likely overlapped with the broadened Ti─O peak at around 790 cm^−^^1^. Due to the complex bonding environment of Ce within the TiO_2_ lattice and the broad nature of the Ti─O peak, isolating the Ce─Ti─O vibration using FTIR alone proved challenging [51, 52]. The band observed around 1550 cm^−^^1^ in the FTIR spectra corresponds to the residual bending vibrations of surface‐adsorbed moisture (H─O─H) and coordinated phytochemical carbonyl/aromatic residues from the plant extract that remain interacting with the metal oxide surface after calcination.

XPS survey scans confirmed the presence of elemental carbon across all samples (Figure 2A). The survey scan revealed the presence of Ti, O, and C, which are attributed to residual organic moieties and coordinating capping residues derived from the *Syzygium cumini* L. plant extract persisting on the NPs' surfaces (Figure 3A). The binding energies of the Ti 2p_3/2_ and Ti 2p_1/2_ peaks at 459.15 and 464.85 eV, respectively (Figure 3B), correspond to Ti^4+^ in the TiO_2_ lattice [53]. The O 1s peak observed across all spectra (Figure 3C) can be attributed to lattice oxygen (O^2^^−^), oxygen within O─Ti─O bonds, and potentially oxygen associated with dopants (Ti─O─Ce and Ti─O─S) [49, 50, 51, 53, 54].

**FIGURE 3:**
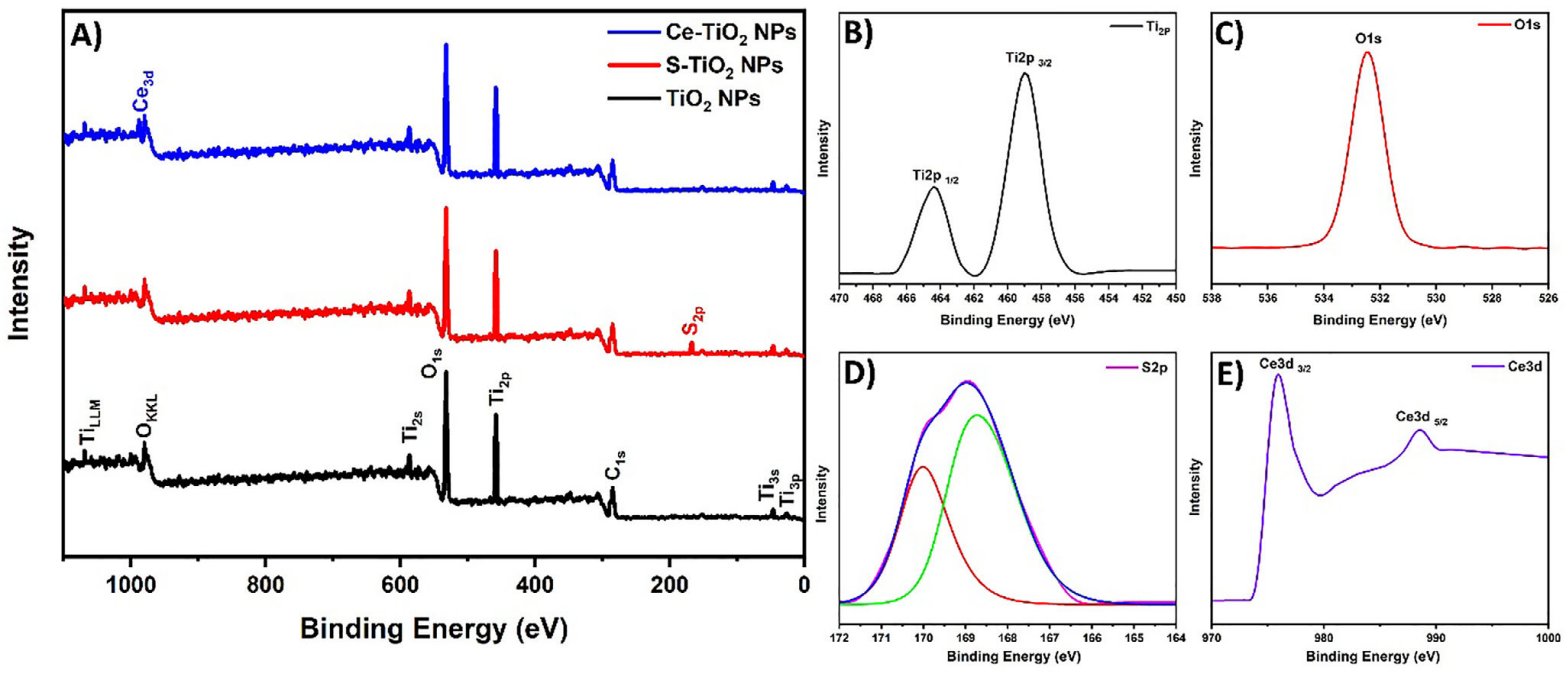
Full survey XPS spectra of TiO_2_ NPs, S─TiO_2_ NPs, and Ce─TiO_2_ NPs (A), high‐resolution XOS spectra of Ti_2P_ (B), O1s (C), S_2P_ (D), and Ce3d (E).

S‐doping in the S─TiO_2_ NPs was confirmed by additional peaks in the S_2p_ spectrum at 170.0 and 168.0 eV (Figure 3D). These binding energies are characteristic of sulfur species coordinated with Ti^4^^+^, suggesting the incorporation of sulfur atoms into the TiO_2_ lattice, potentially as sulfate (SO_4_^2^^−^) or sulfide (S^2^^−^) ions [47, 48, 53]. The Ce─TiO_2_ NPs spectrum (Figure 2E) exhibited peaks corresponding to Ce^3^^+^, known to display a dominant spin‐orbit doublet in XPS [55]. Additionally, the presence of satellite peaks indicates unpaired electrons in Ce^3^^+^, which can induce electronic transitions and contribute to spectral complexity [52]. The prominent peaks for Ce^3^^+^ 3d_3/2_ and Ce^3^^+^ 3d_5/2_ were identified at binding energies of 975 and 998 eV, respectively. The confirmation of sulfur species coordinated with Ti^4+^ and the presence of Ce^3+^ oxidation states with associated satellite peaks highlight distinct electronic perturbations. Sulfur incorporation introduces localized defect states and mid‐gap energy levels that narrow the band gap, whereas cerium introduces redox‐active Ce^3+^/Ce^4+^ sites capable of acting as effective electron traps. Together, these surface and lattice modifications suppress charge‐carrier recombination, enhance light absorption, and synergistically drive improved photocatalytic activity.

Photoluminescence (PL) properties of S and Ce─TiO_2_ NPs were studied to understand their improved photocatalytic efficiency. PL, a well‐suited technique, enables exploration of the recombination rate of photoexcited electron‐hole pairs (e^−^‐h^+^ pairs), directly correlating with photocatalytic performance. The PL emission spectra of pristine TiO_2_, S─TiO_2_, and Ce─TiO_2_ NPs were meticulously recorded upon excitation at 325 nm, as shown in Figure 4A. The PL spectra showed three emission peaks at 410, 460, and 520 nm. The 410 nm peak is attributed to the free exciton emission within the band gap [56]. In contrast, the peaks at 461 and 520 nm likely emanate from defect centers associated with oxygen vacancies, capturing one and two trapped electrons, respectively [57, 58]. The PL intensity of all doped TiO_2_ NPs decreased compared to pure TiO_2_, indicating the dopants' role in impeding *e^−^‐h^+^* pair recombination. The decreased PL emission underscores the synergistic effect between Ti and S or Ce ions, which significantly curtails *e^−^‐h^+^* pair recombination, thereby augmenting the photocatalytic activity of TiO_2_. The enhanced separation of *e^−^‐h^+^* pairs facilitated by S and Ce ions can be attributed to their effective trapping of conduction electrons within TiO_2_, owing to their high reduction potentials (*S^2+^ + 2e^−^ ⇌ S, E^0^ = −0.48 V; Ce^4+^ + e^−^ ⇌ Ce^3+^, E^0^ = +1.72 V*). This phenomenon induces p‐type conductivity within TiO_2_, promoting the formation of a p‐n junction, thereby facilitating the migration of holes toward the photocatalyst surface [59, 60, 61]. The synthesized S─TiO_2_ NPs and Ce─TiO_2_ NPs were subjected to Raman spectroscopy to assess the impact of S and Ce ion doping (Figure 4B). Both S─TiO_2_ NPs and Ce─TiO_2_ NPs samples exhibited characteristics indicative of the anatase phase, displaying six Raman‐active modes: *A_1g_ + 2B_1g_ + 3E_g_* [62]. Raman spectroscopy is highly sensitive to structural alterations, detecting subtle changes such as lattice deformations, which can result in shifts in frequency, broadening of Raman lines, or the emergence of new lines in the spectrum [57]. In all doped TiO_2_ samples, a distinct and intense peak at 144 cm^−1^ corresponding to symmetric lattice angular vibrations of anatase TiO_2_ was observed [54]. Furthermore, a notable reduction in Raman vibrational peak intensity was observed due to the presence of dopants. This reduction can be attributed to modifications in crystallite grain structure induced by S and Ce doping, leading to increased internal stress and subsequent suppression of lattice vibration, resulting in weakened lattice scattering [54]. Specifically, the B_1g_ peak represents the symmetric bending vibration of O─Ti─O, while the A1g peak corresponds to the antisymmetric bending vibration of O─Ti─O [58, 63]. The observed Raman shift of the E_g_(3) mode in S and Ce ion‐doped nanoparticles indicated lattice disorder and oxygen deficiencies resulting from the doping of S and Ce ions; notably, the E_g_(3) mode of anatase is sensitive to Ce doping compared to S doping [64].

**FIGURE 4:**
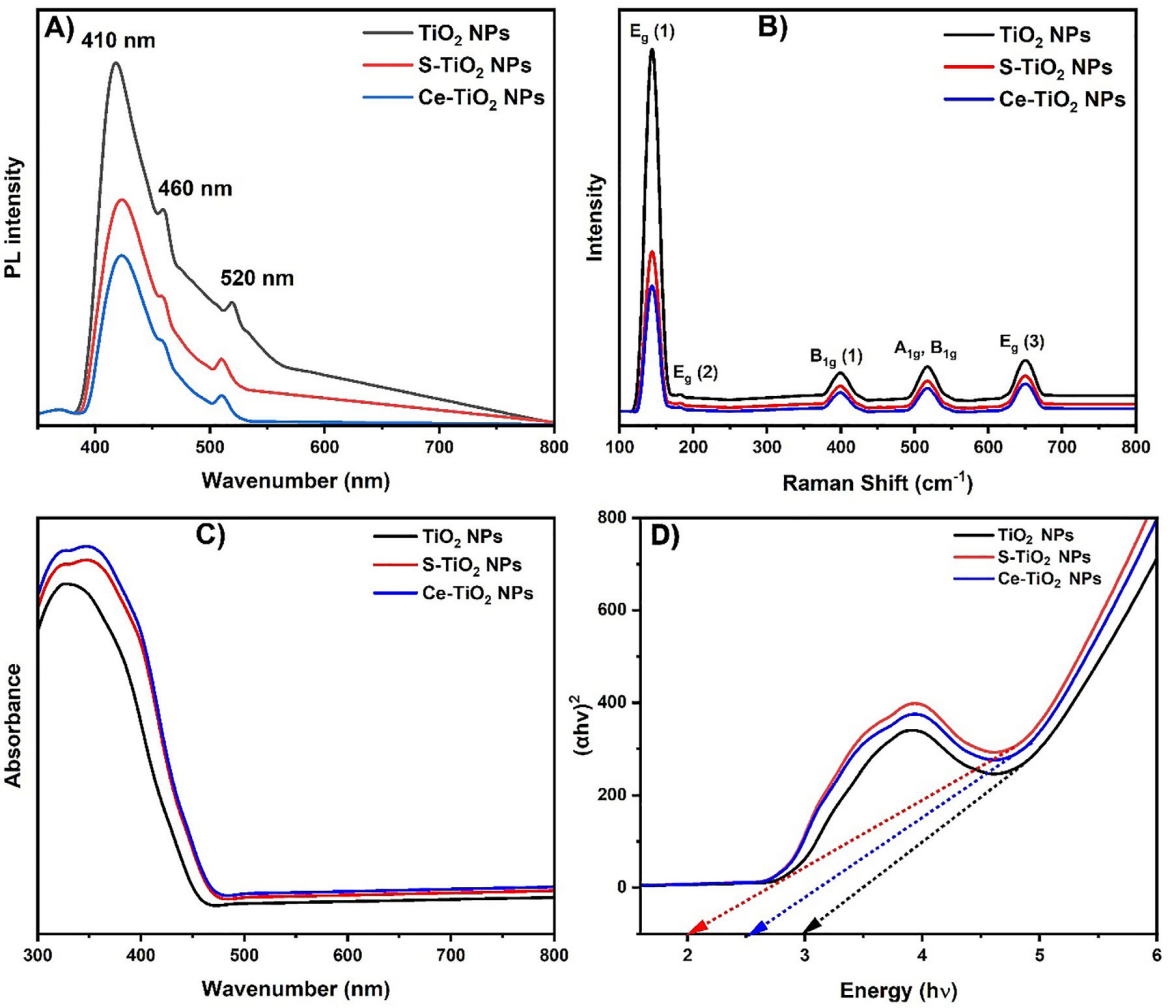
PL emission spectra (A), Raman spectra (B), UV–visible absorbance spectra (C), and Tauc plot (D) of TiO_2_ NPs, S─TiO_2_ NPs, and Ce─TiO_2_ NPs (with dashed lines indicating the linear extrapolations for fundamental band gap and defect‐mediated sub‐band transitions).

Understanding the light absorption properties of materials is pivotal for enhancing their performance in photocatalytic applications. The UV–visible (UV–vis) absorption spectra of pure TiO_2_ nanoparticles (NPs) alongside S─TiO_2_ and Ce─TiO_2_ NPs were investigated to unravel their photoabsorption characteristics (Figure 4C). Pure TiO_2_ NPs exhibited absorption exclusively within the ultraviolet (UV) range due to their substantial band gap energy. This limitation confines their activity to UV light, restricting their effectiveness under visible light irradiation. Incorporating S and Ce ions through doping altered the light absorption behavior of TiO_2_ NPs.

Both S─TiO_2_ and Ce─TiO_2_ NPs displayed a noticeable red shift in their absorption edges compared to pure TiO_2_, signifying an extension of light absorption into the visible spectrum. S and Ce doping introduced new energy levels within the TiO_2_ crystal lattice, positioned between the valence and conduction bands. This narrowing of the band gap energy facilitated the excitation of electrons by visible light, as observed in the redshift [65, 66]. The redshift observed in S─TiO_2_ can be attributed to the creation of new energy levels within the band gap, enabling electron excitation from the valence band of TiO_2_. Similarly, in Ce─TiO_2_, part of the redshift can be ascribed to electron excitation from the ground state of cerium oxide to the Ce 4f energy level [67]. For the band‐gap calculation, the Tauc relation was used, which is based on the following equation:

$$\;{\left(ahv\right)}^{2}=A\left(h\nu \;-\;E_{g}\right)$$

where α is the absorption coefficient, *hν* is the photon energy, *E_g_* is the band‐gap energy, *A* is a constant, and *n* depends on the type of electronic transition. For direct allowed transitions, *n = 2*. The (αhν)^2^ versus hν (photon energy) plot was extrapolated along the linear portions corresponding to both fundamental band edges and defect‐mediated sub‐band transitions to determine the band‐gap and impurity energy levels (E_g_) [57, 63]. The band gap energies of TiO_2_, Ce─TiO_2_, and S─TiO_2_ were estimated and found to be 3.0, 2.5, and 2.0 eV, respectively (Figure 4D). This band‐gap narrowing is a strategic design feature intended to enhance the material's photocatalytic responsiveness across the solar spectrum. While our performance assessment utilized broad‐spectrum light, the engineered electronic structure of the doped nanoparticles allows them to efficiently harvest visible light, a significant departure from the UV‐limited performance of pristine TiO_2_. Control photolysis experiments confirmed that the observed degradation is primarily photocatalytic rather than light‐induced. Consequently, the superior degradation kinetics observed in S and Ce─TiO_2_ systems, even under broad‐spectrum conditions, are directly attributable to the dopant's ability to extend the photocatalytic activity beyond the UV range, effectively minimizing the constraints typically associated with conventional, UV‐dependent photocatalysts.

SEM was employed to investigate the morphology and size distribution of the prepared TiO_2_ NPs, S─TiO_2_ NPs, and Ce─TiO_2_ NPs. All samples exhibited predominantly spherical morphology, as evident from the SEM micrographs (Figure 5A–C). The pure TiO_2_ NPs displayed the most uniform and well‐defined spherical shape among the three (Figure 5A). Doping with S and Ce resulted in a slight disruption of the spherical morphology for S─TiO_2_ NPs (Figure 5B) and a more pronounced disruption for Ce─TiO_2_ NPs (Figure 5C). The nanoparticles size distribution was determined using ImageJ software for image analysis of the SEM micrographs. The particle size for TiO_2_ NPs ranged from 34 to 50 nm, mainly within the 40–42 nm range (Figure 5D). S‐doping slightly increased particle size, with the distribution ranging from 35 to 65 nm and most particles in the 40–45 nm range (Figure 5E). Ce‐doping caused the largest increase in particle size, with the distribution spanning 40 to 65 nm and a peak centered around 45–50 nm (Figure 5F).

**FIGURE 5:**
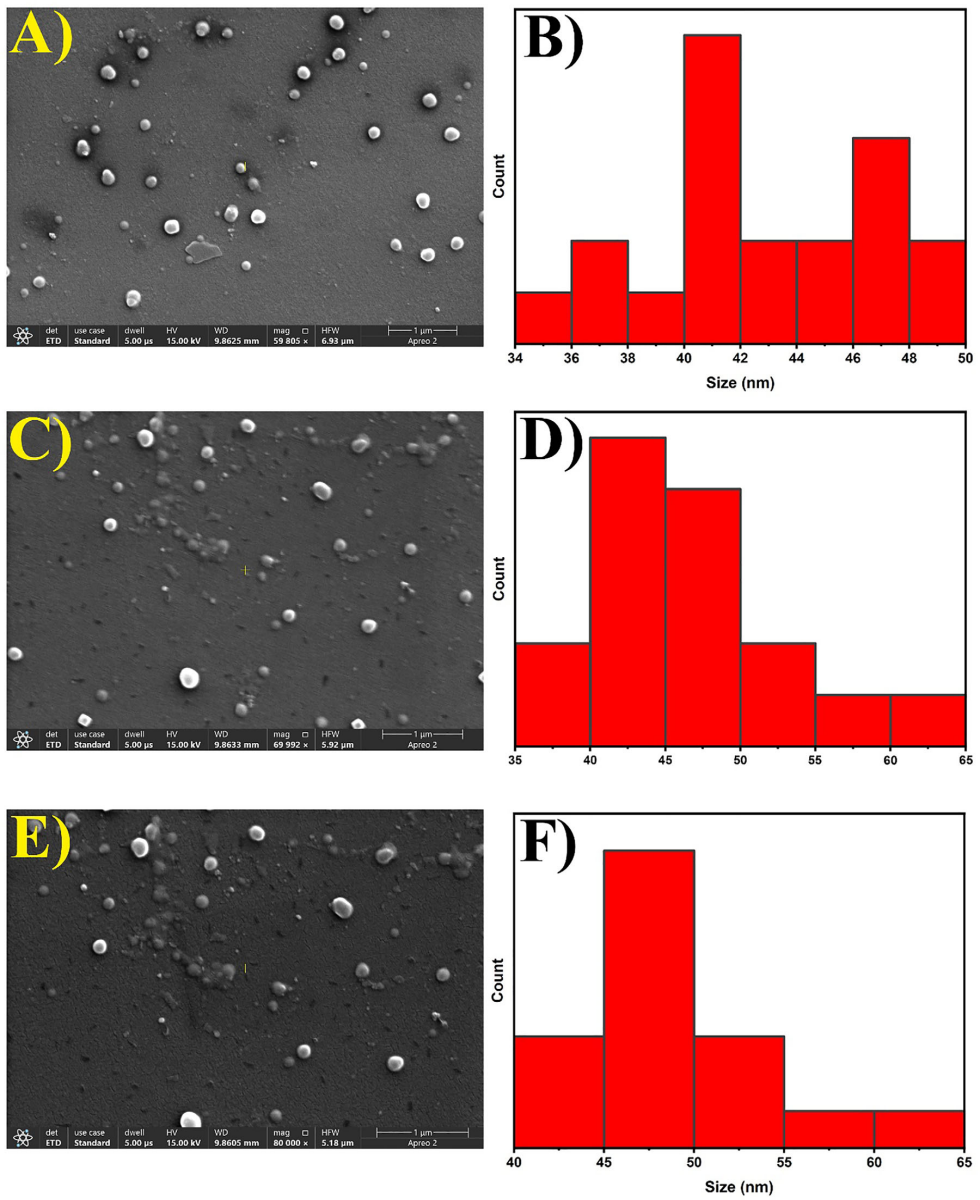
SEM micrographs and particle size histograms of TiO_2_ NPs (A, B), S─TiO_2_ NPs (C, D), and Ce─TiO_2_ NPs (E, F).

### 3.2 Photocatalytic Degradation of RhB, OMZ, and CPS

The photocatalytic efficacy of S─TiO_2_ and Ce─TiO_2_ NPs was assessed for the degradation of various organic pollutants, including RhB, OMZ, and CPS. For each test, a concentration of 20 mg/L of the synthesized NPs was combined with 20 ppm solutions of RhB, OMZ, or CPS. These mixtures were stirred in the dark for 30 min to establish adsorption–desorption equilibrium between the pollutants and the photocatalysts. Next, the solutions were irradiated under the specified conditions, with continuous shaking to ensure uniform exposure; photocatalytic experiments were conducted in triplicate to ensure reliable results. After irradiation, 2 mL aliquots were withdrawn from each solution at 10 min intervals and centrifuged to remove any suspended photocatalyst particles. The supernatant was then analyzed using a UV–vis spectrophotometer to track pollutant degradation. The photocatalytic efficiency of S─TiO_2_ NPs was observed to be slightly higher than that of Ce─TiO_2_ NPs due to the difference in their dopants, as evidenced by the following results, where the degradation percentages of selected pollutants were marginally higher in the case of S─TiO_2_ NPs compared to Ce─TiO_2_ NPs.

The results indicated that S─TiO_2_ NPs exhibited remarkable degradation efficiencies, achieving 98.5% degradation of RhB (Figure 6a), while Ce─TiO_2_ NPs exhibited slightly lower degradation rates. For RhB, Ce─TiO_2_ NPs achieved 97.3% degradation (Figure 6d). In both cases, pH was maintained at pH 10 with a catalyst loading of 20 mg/ppm. The relationship between C/C_0_ and time for RhB for S─TiO_2_ and Ce─TiO_2_ NPs is depicted in Figure 6b,e, respectively. The kinetics and order of the degradation reaction with respect to time were analyzed using the concentration ratio (C/C_0_) and the natural logarithm of the concentration ratio (ln(C/C_0_)) for RhB, respectively, as shown in Figure 6c,f. The exponential decrease in the concentration ratio indicates first‐order reaction kinetics.

**FIGURE 6:**
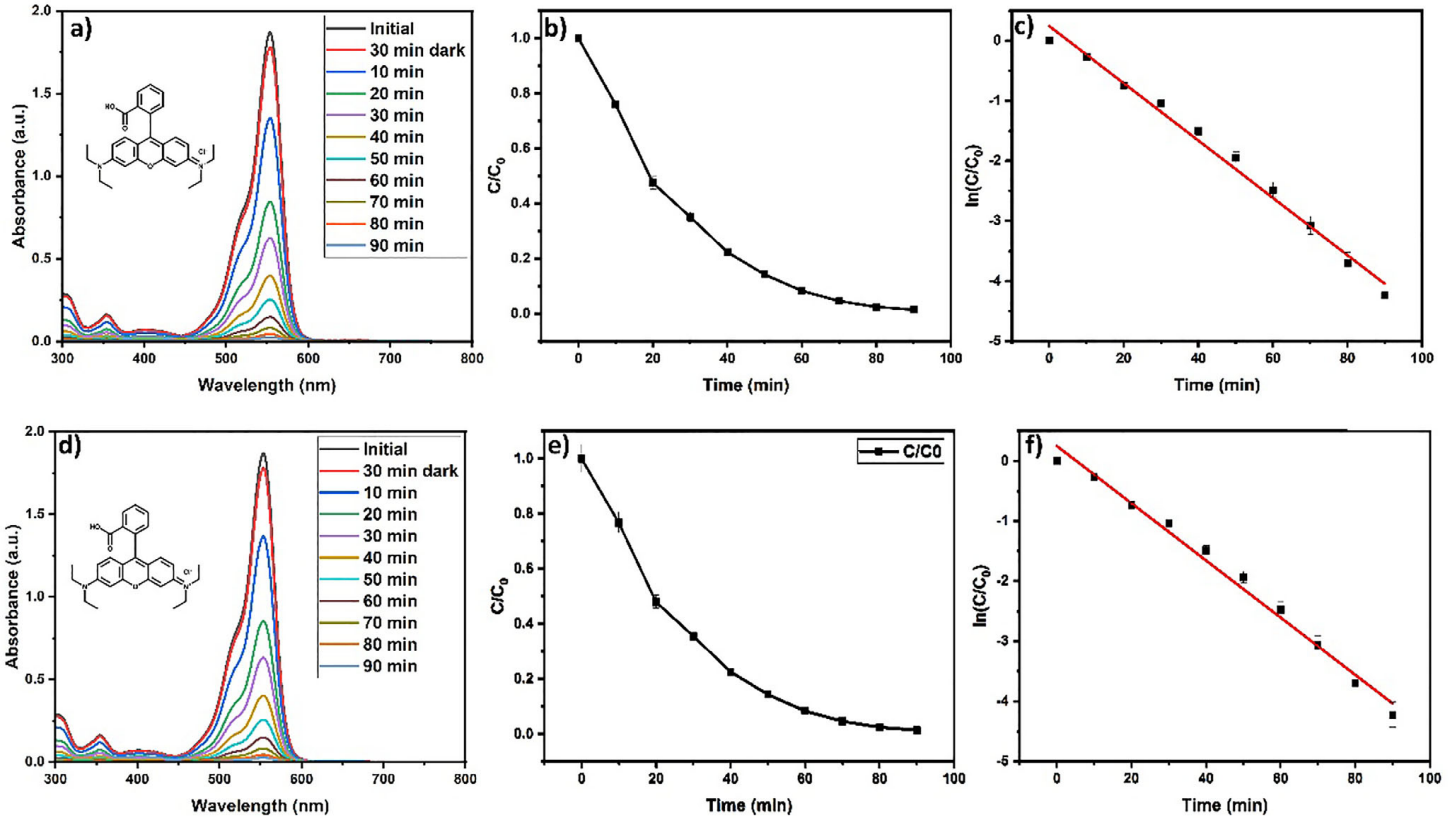
UV–vis spectra illustrating the photocatalytic degradation of RhB with S─TiO_2_ NPs (a) and Ce─TiO_2_ NPs (d), accompanied by their respective kinetics analysis: C/C_0_ plots (b, e) and ln(C/C0) plots (c, f) over time.

Similarly, OMZ degradation reached 96% using S─TiO_2_ NPs (Figure 7a) and 95.55% using Ce─TiO_2_ NPs (Figure 7d) under 20 ppm catalyst loading, pH 2, and a 30 min reaction time. The relationship between C/C_0_ and time for OMZ for S─TiO_2_ and Ce─TiO_2_ NPs is illustrated in Figure 7b,e, respectively. The kinetics and order of the degradation reaction concerning time were analyzed using the concentration ratio (C/C_0_) and the natural logarithm of the concentration ratio (ln(C/C_0_)) for OMZ, respectively, as shown in Figure 7c,f. The exponential decrease in the concentration ratio indicates first‐order reaction kinetics.

**FIGURE 7:**
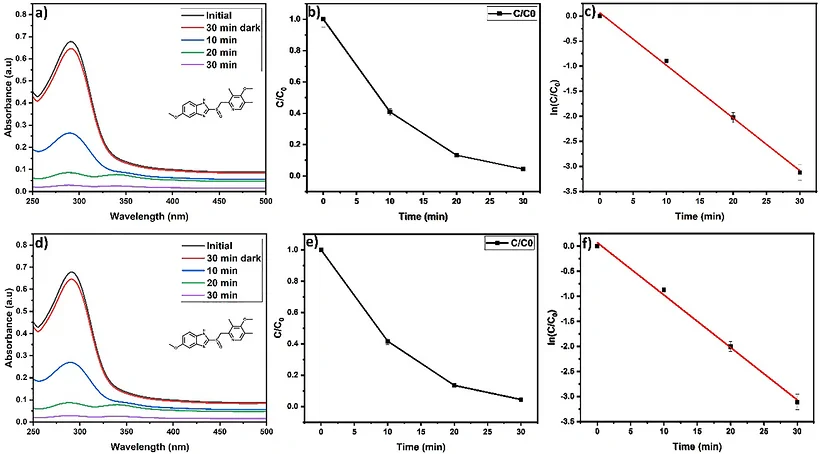
UV–vis spectra illustrating the photocatalytic degradation of OMZ with S─TiO_2_ NPs (a) and Ce─TiO_2_ NPs (d), accompanied by their respective kinetics analysis: C/C_0_ plots (b, e) and ln(C/C_0_) plots (c, f) over time.

CPS degradation was notably high at 98.1% in the case of S─TiO_2_ NPS (Figure 8a) and 97.3% degradation in the case of Ce─TiO_2_ NPs (Figure 8d) with a catalyst loading of 20 mg/ppm, pH 10, and a reaction time of 40 min in both cases. The relationship between C/C_0_ and time for CPS for S─TiO_2_ and Ce─TiO_2_ NPs is presented in Figure 8b,e, respectively. The kinetics and order of the degradation reaction with respect to time were analyzed using the concentration ratio (C/C_0_) and the natural logarithm of the concentration ratio (ln(C/C_0_)) for CPS, respectively, as shown in Figure 8c,f. The exponential decrease in the concentration ratio indicates first‐order reaction kinetics.

**FIGURE 8:**
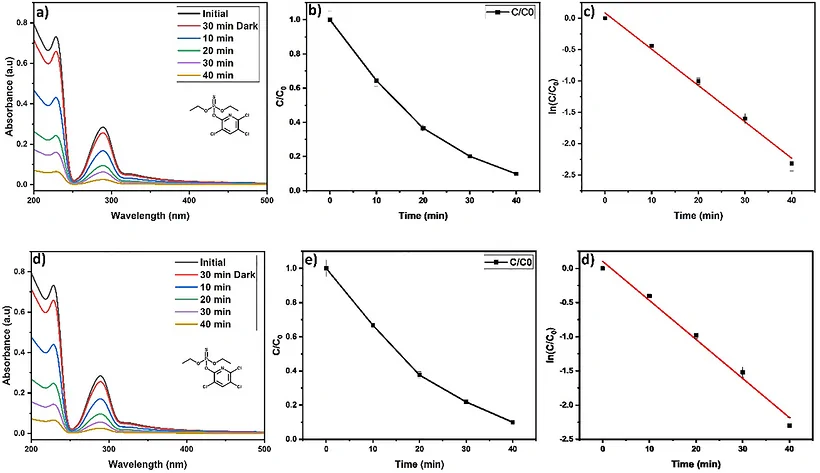
UV–vis spectra illustrating the photocatalytic degradation of CPS with S─TiO_2_ NPs (a) and Ce─TiO_2_ NPs (b), accompanied by their respective kinetics analysis: C/C_0_ plots (c, d) and ln(C/C_0_) plots (e, f) over time.

The choice of dopant significantly influences the photocatalytic performance of TiO_2_ NPs. S─TiO_2_ NPs consistently outperformed Ce─TiO_2_ NPs across all analytes, as summarized in Table 1, which details the calculated pseudo‐first‐order rate constants (*k*). This superior efficiency in S─TiO_2_ is attributed to non‐metallic sulfur doping, which more effectively narrows the band gap, promoting broader visible‐light absorption and enhanced charge‐carrier separation compared to the metallic cerium dopant. Additionally, sulfur introduces lattice defect states that act as active sites for improved pollutant adsorption (Table 1).

**TABLE 1:** The pseudo‐first‐order kinetic rate constants (*k*) and regression coefficients (*R* ^2^) for the photocatalytic degradation of RhB, OMZ, and CPS.

Pollutant	Photocatalyst	Rate constant (k, min^−1^)	*R* ^2^
**RhB**	S─TiO_2_	0.04765	0.988
	Ce─TiO_2_	0.04760	0.986
**OMZ**	S─TiO_2_	0.10497	0.997
	Ce─TiO_2_	0.10468	0.996
**CPS**	S─TiO_2_	0.05799	0.992
	Ce─TiO_2_	0.05714	0.983

### 3.3 Optimization of Degradation Parameters

Various parameters, such as pH, catalyst loading, and reaction time, were optimized to achieve maximum degradation within a short period.

### 3.4 Catalyst Loading

The influence of catalyst loading on the photocatalytic degradation of RhB, OMZ, and CPS was investigated using S─TiO_2_ and Ce─TiO_2_ NPs. The results indicated that increasing catalyst loading initially increased photocatalytic efficiency and pollutant degradation. However, beyond a certain point, further increases in catalyst loading resulted in either a plateau or a decrease in the percentage degradation. This phenomenon can be attributed to the solution turbidity, which impedes the passage of radiation through the solution.

The effect of catalyst loading on RhB, OMZ, and CPS in the case of S─TiO_2_ NPs is shown in Figure 9a, and Ce─TiO_2_ NPs in Figure 9d. The results showed that for S─TiO_2_ NPs, RhB degradation increased from 30.2% to 85% as catalyst loading increased from 10 to 20 mg/ppm catalyst loading increased from 10 to 20 mg/ppm. However, the degradation percentage decreased slightly to higher loadings, reaching 71% at 35 mg/ppm. Similarly, for Ce─TiO_2_ NPs, RhB degradation increased from 25.5% to 84.2% as catalyst loading increased from 10 to 20 mg/ppm but decreased at higher loadings. For OMZ, both S─TiO_2_ and Ce─TiO_2_ NPs showed similar trends, with degradation increasing initially and then decreasing at higher loadings. For S─TiO_2_ NPs, the degradation of OMZ increased from 31.4% to 54.9% with increasing catalyst loading from 10 to 20 mg/ppm, while for Ce─TiO_2_ NPs, it increased from 29.5% to 55.6% over the same range of loadings. Similarly, for CPS degradation, both types of NPs showed increasing degradation percentages with increasing catalyst loading up to a certain point, followed by a decrease or plateau. For S─TiO_2_ NPs, the degradation of CPS increased from 45.7% to 67% with increasing catalyst loading from 10 to 20 mg/ppm, while for Ce─TiO_2_ NPs, it increased from 43.4% to 66.3% over the same range of loadings. These results suggest that the optimal catalyst loading for maximizing photocatalytic efficiency lies within a specific range, beyond that, further increases may not yield significant improvements and could even decrease efficiency due to increased turbidity [68].

**FIGURE 9:**
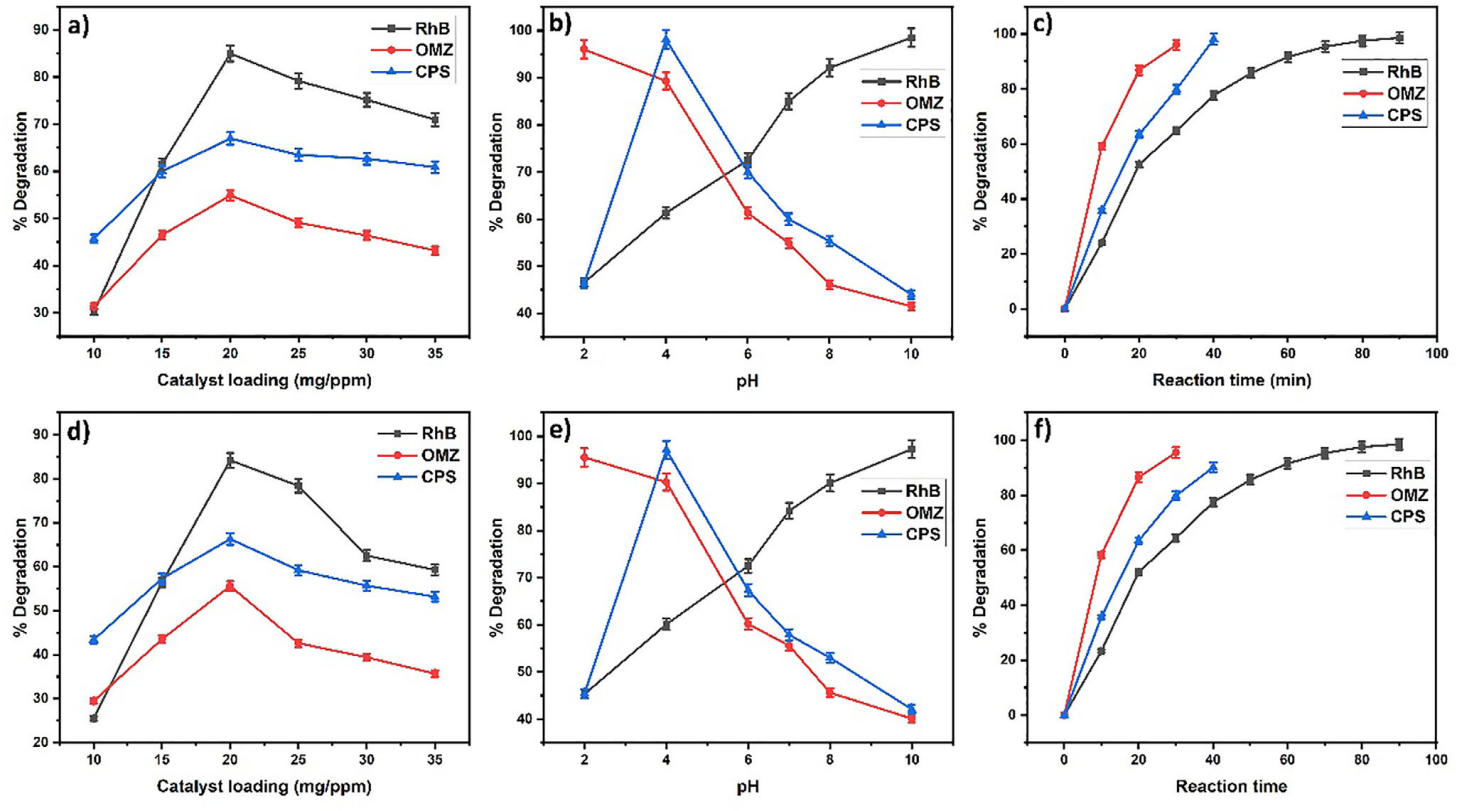
Optimization of degradation parameters for RhB, OMZ, and CPS using S─TiO_2_ and Ce─TiO_2_ NPs: (a–c) show % degradation vs. catalyst loading, pH, and reaction time for S─TiO_2_ NPs, while (d–f) represent the same for Ce─TiO_2_ NPs.

### 3.5 Effect of pH

The degradation rates of RhB (RhB), OMZ (OMZ), and CPS (CPS) were assessed across varying pH levels using both S─TiO_2_ and Ce─TiO_2_ NPs as photocatalysts. The pH of the solution is a critical determinant of the degradation efficiency of these molecules due to its influence on their chemical properties and interactions with the catalyst surface [69].

The effect of pH on the % degradation of RhB, OMZ, and CPS in the case of S─TiO_2_ NPs is shown in Figure 9b, and Ce─TiO_2_ NPs are shown in Figure 9e. For RhB degradation using S─TiO_2_ NPs, the percentage degradation exhibited an upward trend with increasing pH, peaking at 98.5% at pH 10. Similarly, Ce─TiO_2_ NPs achieved a maximum degradation of 97.3% at the same pH level. RhB, below a pH of approximately 4.0, predominantly exists in its protonated form, which is relatively stable and thus exhibits slower degradation rates. However, as the pH surpasses 4.0, RhB deprotonates, forming a negatively charged species that is less stable and degrades faster [70]. The higher degradation rates observed for RhB at elevated pH levels with both S─TiO_2_ and Ce─TiO_2_ NPs can be attributed to the increased prevalence of the deprotonated form of RhB, which is more susceptible to photocatalytic degradation. OMZ, being a weak base, displays enhanced solubility and reactivity under acidic conditions. As the pH decreases, the concentration of H^+^ ions rises, promoting the protonation of OMZ molecules. This protonation alters the chemical characteristics of OMZ, rendering it more susceptible to degradation [71]. Consequently, acidic pH environments foster the efficient degradation of OMZ. Specifically, in OMZ degradation, S─TiO_2_ NPs showcased superior degradation efficacy at lower pH levels, achieving a peak degradation of 96% at pH 2. Conversely, Ce─TiO_2_ NPs exhibited marginally lower degradation rates, achieving a maximum of 95.55% at the same pH level. CPS displays variable degradation rates depending on pH, attributable to its dual existence in distinct forms. CPS predominantly adopts its neutral form at medium pH levels, rendering it more prone to degradation. Conversely, as pH levels rise, CPS transitions into its ionized form, which exhibits greater stability and consequently degrades at a slower pace. This phenomenon may be attributed to repulsion between the negatively charged photocatalyst surface and hydroxide anions at higher pH levels, which impedes the formation of OH · radicals and thereby diminishes photocatalytic degradation efficiency [72]. Both S─TiO_2_ and Ce─TiO_2_ NPs exhibited analogous trends in CPS degradation, reaching peak efficiency at pH 4. Specifically, S─TiO_2_ NPs achieved a degradation efficiency of 98.1%, while Ce─TiO_2_ NPs attained 97.1% efficiency at this pH level. CPS's distinctive pH‐dependent degradation behavior underscores the critical role of pH optimization in maximizing the efficacy of photocatalytic pollutant degradation processes, highlighting the necessity of tailored pH conditions for enhanced performance. These findings underscore the significant influence of pH on the photocatalytic degradation of RhB, OMZ, and CPS using S─TiO_2_ and Ce─TiO_2_ NPs as catalysts. Further investigation is warranted to elucidate the underlying mechanisms and optimize pH conditions for specific pollutant degradation processes, emphasizing the need for tailored approaches to enhance the efficiency of photocatalytic pollutant degradation.

### 3.6 Effect of Reaction Time

The influence of reaction time on the degradation of RhB, OMZ, and CPS was investigated using both S─TiO_2_ and Ce─TiO_2_ NPs as photocatalysts. The effect of reaction time on % degradation of RhB, OMZ, and CPS in the case of S─TiO_2_ and Ce─TiO_2_ NPs is shown in Figure 9c,f, respectively. For RhB degradation with S─TiO_2_ NPs, the percentage degradation increased progressively with longer reaction times, reaching a maximum of 98.558% after 90 min. Similarly, Ce─TiO_2_ NPs exhibited a maximum degradation of 98.544% at the same time point. RhB degradation rates accelerated with longer reaction times, indicating that prolonged photocatalytic exposure enhances the degradation process. For OMZ degradation using S─TiO_2_ NPs, the percentage degradation increased from 59.141% at 10 min to 96% at 30 min. Similarly, Ce─TiO_2_ NPs exhibited a maximum degradation of 95.551% at 30 min. The degradation rates of OMZ show a rapid increase within the initial 30 min, suggesting that the degradation process nears completion within this timeframe. For CPS degradation using S─TiO_2_ NPs, the percentage degradation increased steadily from 35.673% at 10 min to 98.1% at 40 min. Ce─TiO_2_ NPs exhibited a similar trend, with a maximum degradation of 97.3% at 40 min. The degradation rates of CPS increased gradually with longer reaction times, indicating a continuous enhancement of the degradation process over time. These results demonstrate the significant impact of reaction time on the photocatalytic degradation of RhB, OMZ, and CPS using S─TiO_2_ and Ce─TiO_2_ NPs as catalysts. Prolonged reaction times result in higher degradation efficiencies, suggesting that extended exposure to photocatalysis enhances the degradation process by allowing more interactions between the pollutants and the photocatalyst surface [73]. The findings highlight the importance of optimizing reaction times to achieve maximum pollutant degradation efficiency in photocatalytic treatment processes.

### 3.7 Reusability Assessment of S─TiO2 and TiO2 NPs

In the reusability test both S─TiO_2_ and Ce─TiO_2_ nanoparticles were evaluated for their ability to maintain degradation efficiency over multiple cycles across all analytes, including RhB, OMZ, and CPS. For S─TiO_2_ nanoparticles, the degradation percentages of RhB remained consistently high, ranging from 98.5% in the first cycle to 95% in the 21st cycle. Similarly, OMZ degradation percentages ranged from 96% to 94% over the same cycles, while CPS degradation percentages ranged from 98.1% to 96.3%, as shown in Figure 10a. In the case of Ce─TiO_2_ nanoparticles, the degradation percentages of RhB ranged from 97.3% to 94.8%, while OMZ degradation percentages ranged from 96% to 95.2%. CPS degradation percentages ranged from 95.55% to 93.7% over the evaluated cycles, as shown in Figure 10b. S─TiO_2_ and Ce─TiO_2_ nanoparticles demonstrated relatively stable degradation efficiencies across multiple cycles for all analytes, indicating their potential for repeated use in photocatalytic degradation applications across various organic pollutants [74]. While post‐cycling PXRD and XPS analyses were not performed, the high retention of photocatalytic efficiency over successive cycles confirms the structural stability and robustness of the catalysts. These results collectively highlight the feasibility of employing S─ and Ce─TiO_2_ nanoparticles in practical environmental remediation scenarios where long‐term performance and reusability are essential considerations.

**FIGURE 10:**
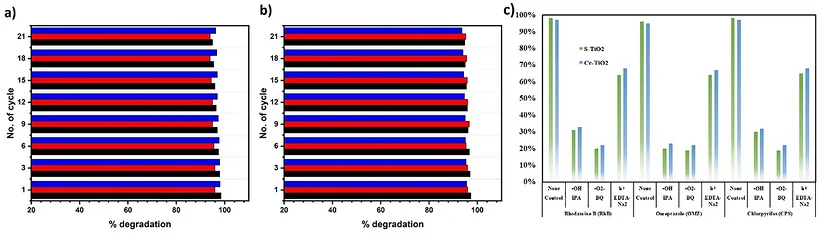
% degradation vs the number of catalytic cycles of (a) S─TiO_2_ NPs, (b) Ce─TiO_2_ NPs (black bar: RhB, red bar: OMZ, and blue bar: CPS), and (c) radical scavenger study.

### 3.8 Radical Scavenging Study and Photocatalytic Mechanism

To elucidate the underlying mechanism and identify the predominant reactive oxygen species governing the photocatalytic degradation of RhB, OMZ, and CPS, control experiments using specific radical scavengers were performed under optimized conditions. Isopropyl alcohol (IPA) was introduced as a selective scavenger for hydroxyl radicals (•OH) Benzoquinone (BQ) was utilized to trap superoxide radicals (•O_2_^−^). Disodium EDTA (EDTA‐Na_2_) was added to scavenge photogenerated valence band holes (h^+^). Adding BQ and IPA dramatically suppressed degradation efficiency across all three target pollutants (Figure 10c). Specifically, when BQ was introduced, the removal efficiencies of RhB, OMZ, and CPS dropped sharply from over 95% down to 20%–30%, confirming that O^2−^ acts as the primary oxidizing species. Similarly, the addition of IPA caused a substantial decrease in degradation performance, indicating that •OH also plays a critical role in the oxidative degradation pathway. The introduction of EDTA‐Na_2_ led to a moderate reduction in degradation rates, demonstrating that photogenerated holes contribute directly to oxidation or assist in secondary radical generation. These findings confirm that sulfur and cerium doping successfully narrows the band gap and suppress electron‐hole recombination, thereby facilitating the robust generation of •O_2_^−^ and •OH radicals responsible for the exceptional photocatalytic activity of S─TiO_2_ and Ce─TiO_2_ nanoparticles.

### 3.9 Comparative Analysis of S─TiO2 and TiO2 NPs With Reported Photocatalyst

The comparative analysis of the green‐synthesized S─TiO_2_ NPs and Ce─TiO_2_ NPs, along with other reported photocatalysts for degrading RhB, CPS, and OMZ, reveals several important insights into their performance and applicability (Table 2). A previous study used TiO_2_ NPs, employed a high catalyst loading of 500 mg/L, and achieved a moderate RhB degradation of 95.38% in 40 min [75, 76, 77]. However, such a high dose of catalyst may not be practical on a large scale, and additional ultrasonic energy was required, suggesting limitations in the efficiency of the process. Another material, g‐C_3_N_4,_ achieved a similar % degradation percentage of 95% for RhB but required a significantly longer degradation time of 320 min. Moreover, degradation efficiency decreased to 92.5% after five cycles, indicating potential limitations in catalyst reusability. MMT/TiO_2_ achieved complete degradation (100%) of RhB in 60 min with a low catalyst loading of 10 mg [78]. However, the complex synthesis protocol of the photocatalyst might hinder its scalability and practical application. CdS NRs exhibited a moderate degradation percentage of 88.4% for RhB in 120 min with a catalyst loading of 10 mg [79]. Despite the relatively low degradation efficiency and high degradation time, this photocatalyst demonstrated potential for further optimization. The current work utilizing S─TiO_2_ and Ce─TiO_2_ NPs achieved superior degradation efficiencies of 98.1% and 97.3% for RhB in just 90 min with a catalyst loading of 20 mg. Notably, these green‐synthesized nanoparticles offer non‐toxic synthesis routes and demonstrate high degradation efficiency quickly, making them promising candidates for practical applications.

**TABLE 2:** Comparative analysis of the synthesized materials with already reported materials from the literature.

Material	Catalyst loading	Degradation time	Degradation (%)	Pollutant	Concentration	Comments	References
TiO_2_ NPs	25 mg	40 min	95.38%	RhB	20 ppm	Low % degradation, high dose of catalyst, use of additional ultrasonic energy.	[75, 86]
g‐C_3_N_4_	300 mg	320 min	95%	RhB	—	Low % degradation decreased to 92.5% in 5 cycles, long degradation time.	[76]
MMT/TiO_2_	10 mg	60 min	100%	RhB	3 ppm	Complex synthesis protocol of photocatalyst	[77, 87]
CdS NRs	10 mg	120 min	88.4%	RhB	10 ppm	Low % degradation, high degradation time	[78]
S─TiO_2_ NPs	20 mg	90 min	98.1%	RhB	20 ppm	Non‐toxic green synthesis, high degradation % in a short time and catalytic cycles	Current work
Ce─TiO_2_ NPs	20 mg	90 min	97.3%	RhB	20 ppm	Non‐toxic green synthesis, high degradation % in short time and catalytic cycles	Current work
nHAP@CFGO/ZnR	10 mg	30 min	100%	CPS	5 ppm	Complex synthesis, long reaction steps, several toxic reagents, tested up to five cycles only	[88]
Th^4+^ doped TiO_2_	150 mg	45 min	100%	CPS	—	High catalyst loading, no reusability test, concentration of CPS not specified	[89]
CoFe_2_O_4_@TiO_2_/rGO	20 mg	60 min	100%	CPS	5 ppm	Complex and toxic synthesis, high catalyst loading	[84]
TiO_2_ NPs	37.5 mg	120 min	80%	CPS	30 ppm	Long reaction time, high catalyst loading, low % degradation, no reusability tests	[90]
Ag@Citrate NPs	25 mg	180 min	100%	CPS	4 ppm	Unclear catalyst dosage, high degradation time, no reusability tests	[82]
S─TiO_2_ NPs	300 mg	40 min	96% 98.1%	CPS	1 ppm	Non‐toxic green synthesis, high degradation % in a short time and catalytic cycles	Current work
Ce─TiO_2_ NPs	10 mg	40 min	97.3%	CPS	1 ppm	Non‐toxic green synthesis, high degradation % in short time and catalytic cycles	Current work
Y_3_NbO_7_	10 mg	64 min	89%	OMZ	20 ppm	High reaction time, high band gap, lack of degradation efficiency (%) and no catalytic cycles	[83]
rGO/Ag@ZnO	20 mg	30	96%	OMZ	1 ppm	Complex and lengthy material synthesis, using toxic reagents, only five catalytic cycles	[89, 91, 92]
S─TiO_2_ NPs	20 mg	30 min	96%	OMZ	20 ppm	Non‐toxic green synthesis, high degradation % in short time and catalytic cycles	Current work
Ce─TiO_2_ NPs	20 mg	30 min	95.55%	OMZ	20 ppm	Non‐toxic green synthesis, high degradation % in short time and catalytic cycles	Current work

Previous studies on the degradation of CPS have utilized several materials such as nHAP@CFGO/ZnR, Th^4+^ doped TiO_2_, CoFe_2_O_4_@TiO_2_/rGO, TiO_2_ NPs, and Ag@Citrate NPs [72, 79, 80, 81, 82], utilized various photocatalysts with differing degrees of synthesis complexity and achieved complete degradation. However, some of these methods involved toxic reagents and complex synthesis steps, limiting their practicality and scalability. The current work with S─TiO_2_ and Ce─TiO_2_ NPs achieved remarkable degradation efficiencies of 96% and 97.3% for CPS in 40 min with a catalyst loading of 20 mg. Moreover, these nanoparticles were synthesized using non‐toxic green routes and demonstrated high degradation efficiency quickly, indicating their potential for practical applications.

The degradation of OMZ has been reported with a limited number of photocatalysts such as Y_3_NbO_7_ [83] and rGO/Ag@ZnO [71, 84, 85], having varying degrees of efficiency and complexity such as high reaction time, high band gap, lack of degradation efficiency (%) and no catalytic cycles or a limited number of catalytic cycles showing poor reusability of the photocatalysts while the current work with S─TiO_2_ and Ce─TiO_2_ NPs achieved high degradation efficiencies of 96% and 95.55%, respectively, for OMZ in just 30 min with a catalyst loading of 20 mg. These green‐synthesized nanoparticles offer promising results with non‐toxic synthesis routes and high degradation efficiency within a short time, highlighting their potential for practical applications. Comparative analysis underscores the superior performance and practical advantages of the green‐synthesized S─TiO_2_ and Ce─TiO_2_ NPs for degrading RhB, CPS, and OMZ compared to other reported photocatalysts. These nanoparticles offer high degradation efficiencies, short reaction times, non‐toxic synthesis routes, and potential for reusability, making them promising candidates for environmental remediation applications.

### 3.10 Photocatalytic Degradation Mechanism

The photocatalytic degradation of RhB, OMZ, and CPS by S─TiO_2_ and Ce─TiO_2_ nanoparticles follows a series of key steps, as shown in Scheme 1. The unique properties of sulfur (S) and cerium (Ce) doping play a crucial role in enhancing photocatalytic efficiency by modifying the electronic structure of TiO_2_, improving charge separation, and promoting the generation of reactive oxygen species.

- Adsorption: The process begins with the adsorption of organic pollutants (RhB, OMZ, and CPS) onto the surface of the photocatalyst nanoparticles. This occurs due to attractive interactions between the pollutants and active sites on the catalyst surface. The pollutants become concentrated and immobilized, making them more susceptible to subsequent photodegradation. The S and Ce dopants enhance the catalyst's surface properties, which increase the affinity between the pollutants and the catalyst. This adsorption step can be represented as:
$$\mathrm{Pollutan}t_{\left(\mathrm{aq}\right)}+\mathrm{Catalys}t_{\left(s\right)}\;⇌\;\mathrm{Pollutant}--\mathrm{Catalys}t_{\left(s\right)}$$
- Generation of electron‐hole pairs: Upon exposure to light, the photocatalyst nanoparticles absorb photons with energy equal to or greater than their bandgap energy. This absorption leads to the excitation of electrons from the valence band (*VB*) to the conduction band (*CB*), creating electron‐hole pairs (*e^−^‐h+*) [86]. Doping S─TiO_2_ and Ce─TiO_2_ enhances light absorption, enabling the photocatalyst to utilize both ultraviolet (UV) and visible light. This process can be expressed as:
$$\mathrm{Photocatalys}t_{\left(s\right)}+h\nu \;\to \;{e_{\mathrm{CB}}}^{-}+{h_{V}}^{+}$$
- Redox reactions: The photogenerated electrons (*e^−^*) and holes (*h^+^*) are involved in redox reactions with adsorbed species on the catalyst surface. The photogenerated holes (*h^+^*) are highly oxidizing species (*• OH*) [87]. These hydroxyl radicals are powerful oxidizing agents that initiate the degradation of organic pollutants. These species participate in subsequent redox reactions, as represented below:
$${h_{\mathrm{VB}}}^{+}+H_{2}O\;\to \;•\mathrm{OH}+H^{+}$$
$$2{e_{\mathrm{CB}}}^{-}+O_{2}+2H^{+}\;\to \;H_{2}O_{2}$$
- Degradation of organic pollutants: The hydroxyl radicals (• OH) generated on the surface of the photocatalyst nanoparticles react with the adsorbed organic pollutants, initiating a series of degradation reactions [87]. The specific degradation pathways for each pollutant are as follows:

**SCHEME 1:**
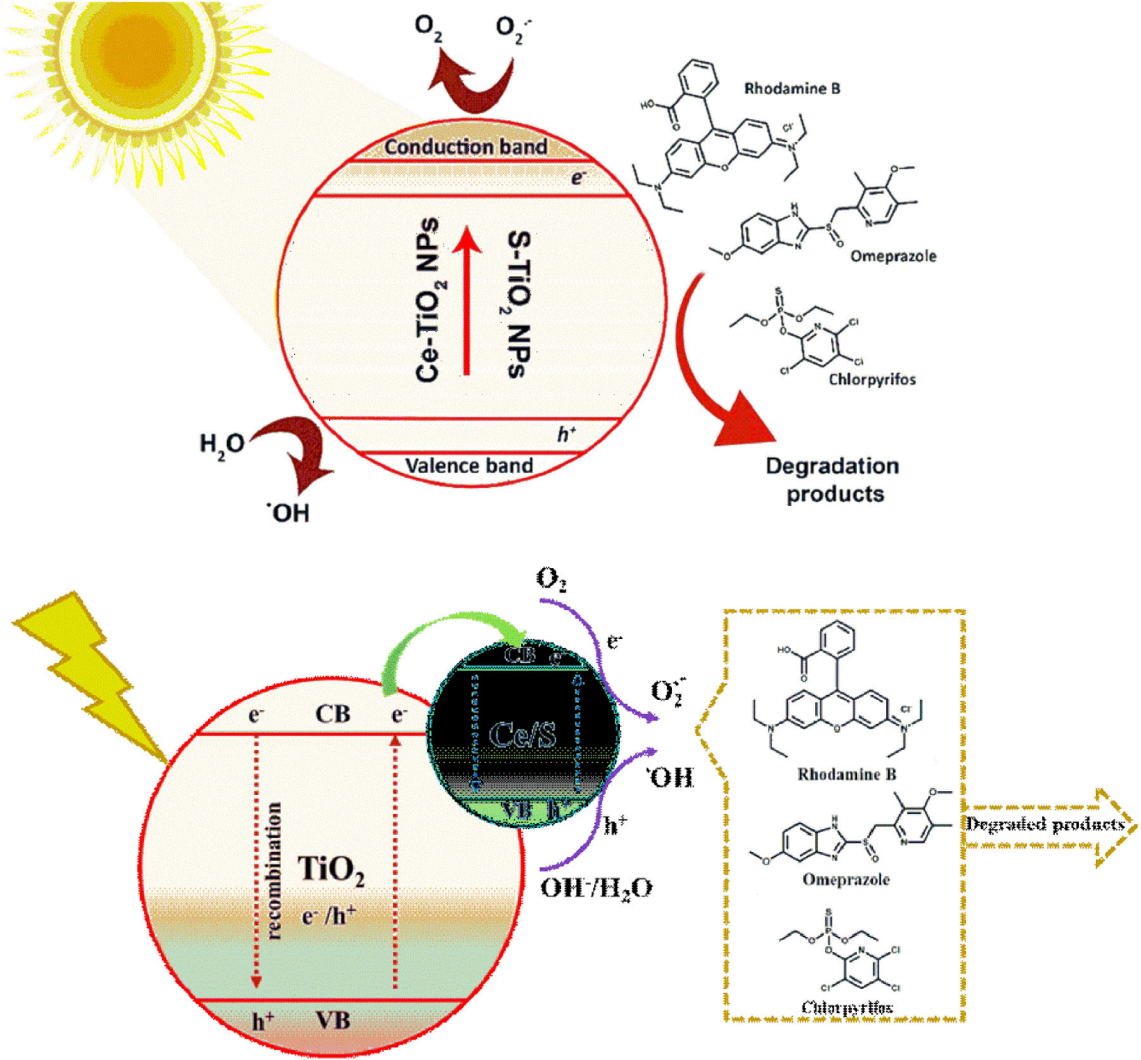
Systematic illustration of photocatalytic degradation of RhB, OMZ, and CPS using S─TiO_2_ NPs and Ce─TiO_2_ NPs.

Hydroxyl radicals (•OH) and superoxide radicals (•O_2_^−^) attack the aromatic structure of RhB, causing the cleavage of its conjugated system. This leads to the mineralization of RhB into simpler, non‐toxic molecules such as CO_2_ and H_2_O. The enhanced oxidative power of the doped TiO_2_ increases the degradation rate. Like RhB, the hydroxyl and superoxide radicals generated by the excited TiO_2_ NPs attack OMZ molecules. This leads to bond cleavage in the aromatic rings and aliphatic chains, forming smaller intermediates, which eventually degrade into non‐toxic by‐products. The radicals attack the pesticide's molecular structure for CPS, breaking the C─Cl and C─P bonds. This degradation pathway produces less toxic products, and further oxidation leads to the mineralization of CPS into harmless by‐products like CO_2_ and H_2_O.

Although both dopants improve photocatalytic performance, they operate via distinct electronic mechanisms. Sulfur acts as a non‐metallic dopant that substitutes into the TiO_2_ lattice, introducing localized mid‐gap energy levels that narrow the band gap, facilitate visible‐light absorption, and promote the generation of reactive sulfate radicals. In contrast, metallic cerium doping primarily functions by suppressing electron‐hole recombination; acting as an electron trap, Ce^3+^ is oxidized to Ce^4+^ during photocatalysis, which enhances the formation of superoxide radicals (Scheme 1).

The degradation reactions typically involve abstracting hydrogen atoms from the organic molecules, forming intermediate products with increased polarity and decreased molecular weight. Subsequent reactions may involve further oxidation, hydroxylation, or fragmentation of the intermediate products, ultimately mineralizing pollutants into harmless by‐products such as water and carbon dioxide. This process can be represented as follows:

RhB+•OH→IntermediateProducts

OMZ+•OH→IntermediateProducts

CPS+•OH→IntermediateProducts

v. Desorption and product release: After degradation, the intermediate products and by‐products desorb from the catalyst surface into the solution, ensuring the continuous availability of active sites for subsequent cycles. Following degradation, intermediate products and by‐products desorb from the catalyst surface into the surrounding solution. This desorption process ensures the continuous availability of active sites on the catalyst surface for subsequent pollutant adsorption and degradation cycles.
vi. Regeneration of catalyst: After the completion of each degradation cycle, the catalyst nanoparticles may undergo regeneration, wherein any surface defects or inactive sites are restored, allowing them to maintain their photocatalytic activity over multiple use cycles. This process allows the catalyst to maintain its photocatalytic activity over multiple cycles, preparing it for further pollutant degradation reactions.

The photocatalytic degradation mechanism involves a series of complex physicochemical processes driven by light‐induced redox reactions on the surface of the semiconductor photocatalysts. The efficiency of the degradation process depends on various factors, including the properties of the catalyst nanoparticles, the nature of the organic pollutants, the intensity and spectrum of the incident light, and reaction conditions such as pH, catalyst loading, and reaction time, which were optimized as described above (Scheme 1).

## 4 Conclusions

In conclusion, green‐synthesized Ce‐ and S‐doped TiO_2_ NPs using *Syzygium cumini* L. leaves were synthesized and characterized using SEM, FT‐IR, UV–visible spectrophotometry, photoluminescence spectroscopy, and x‐ray photoelectron spectroscopy to assess the morphology, structure, and photocatalytic properties of the synthesized NPs. The photocatalytic assessments demonstrated the efficacy of S─TiO_2_ and Ce─TiO_2_ NPs in degrading the target pollutants such as RhB, OMZ, and CPS. S─TiO_2_ NPs exhibited slightly higher degradation efficiencies than Ce─TiO_2_ NPs across all analytes. The optimization of degradation parameters, including catalyst loading, pH, and reaction time, revealed their significant impact on the degradation process. Both S─TiO_2_ and Ce─TiO_2_ NPs demonstrated first‐order kinetics in the degradation of RhB, OMZ, and CPS, with degradation efficiencies reaching up to 98.5% and 97.3% for RhB, 96% and 95.55% for OMZ, and 98.1% and 97.3% for CPS, respectively. The reusability assessment of S─TiO_2_ and Ce─TiO_2_ NPs demonstrated their ability to maintain degradation efficiency over multiple cycles, indicating their potential for practical applications in wastewater treatment. The degradation mechanism elucidated the key steps involved, including adsorption, generation of electron‐hole pairs, redox reactions, degradation of organic pollutants, desorption and product release, and catalyst regeneration. The mechanism highlighted the role of photocatalyst nanoparticles in initiating and facilitating the degradation of organic pollutants under irradiation. This study contributes to the development of efficient photocatalytic systems for environmental remediation.

## Author Contributions

**Fahim Ullah**, is Ph.D. student, carried out all the research work and submitted first draft of the paper; **Jafar Saifullah** and **Sufian Rasheed** helped in part of the work and experimental characterization techniques; **Saima Gul** is the co‐supervisor responsible for execution of research; **Safina Sahrif** helped in parts of the experimental work and moral support to the principal author; **Syed Shakil Abbas** helped in finalizing the draft and discussion of the results; **M.I. Khan** is the Supervisor of the student responsible for successful completion from start to the end.

## Funding

The authors confirm that no funding was received for this research project.

## Conflicts of Interest

The authors declare no conflicts of interest.

## Data Availability

The authors declare that the raw data supporting the findings of this study are available within the paper; and will be made available with the principal as well corresponding author upon reasonable request. Further, all other relevant raw data acquired/generated, analyzed and presented, including experimental, spectroscopic and computational data, are included in this paper.
